# Promoted hydrogen activation and spillover over Pt/Co_3_O_4_ by facet engineering of Co_3_O_4_ for enhanced catalytic hydrogenation

**DOI:** 10.1039/d5sc09402j

**Published:** 2026-03-13

**Authors:** Hui Yun, Jiao Feng, Wanying Peng, Mi Xiong

**Affiliations:** a College of Materials and Chemistry & Chemical Engineering, Chengdu University of Technology Chengdu 610059 China xiongmi@cdut.edu.cn

## Abstract

The exposed facets of supported metal catalysts play a crucial role in catalytic hydrogenation performance. However, the internal relationship between the support crystal facet and catalytic performance needs to be further explored. Herein, a series of well-defined Pt/Co_3_O_4_-x catalysts are fabricated with similar Pt nanoparticle sizes, identical metal loadings, and tailored Co_3_O_4_ crystal facets (x = o, t, c; where “o”, “t”, and “c” denote Co_3_O_4_ exposing predominantly (111), mixed (111)/(100), and (100) facets, respectively). The electronic structure of Pt nanoparticles and the hydrogen spillover capability of Pt/Co_3_O_4_ are modulated by exposing different crystal facets of Co_3_O_4_. For the 4-nitrophenol (4-NP) hydrogenation reaction with H_2_ as the hydrogen source, the Pt/Co_3_O_4_-o catalyst with more Pt^0^ species and stronger hydrogen spillover capability exhibits the best hydrogenation activity with a turnover frequency (TOF) of 164.2 h^−1^. Mechanistic studies indicate that, compared with Pt/Co_3_O_4_-c, the Pt/Co_3_O_4_-o exhibits weaker adsorption and activation of the nitro group, while its ability to activate H_2_ is stronger. The enhanced catalytic activity of Pt/Co_3_O_4_-o is attributed to promoted hydrogen activation and spillover. This work highlights support crystal facet engineering for regulating the electronic structure and hydrogen spillover effect, which provides in-depth insight into catalyst design and hydrogenation mechanism.

## Abstract 2

The (111) facet-induced electron-rich Pt nanoparticles on Co_3_O_4_ accelerate both H_2_ activation and hydrogen spillover dynamics, leading to a superior turnover frequency (TOF) in catalytic hydrogenation.

## Graphical abstract

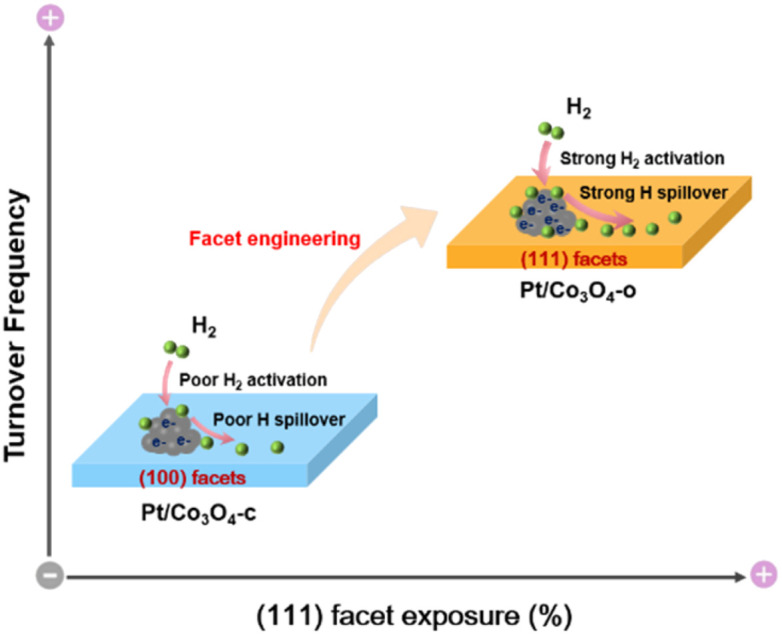

## Introduction

Catalytic hydrogenation plays a pivotal role in environmental protection and industrial processes,^1,2^ particularly for the degradation of persistent organic pollutants such as 4-nitrophenol (4-NP),^3^ a common toxic contaminant in wastewater.^4–6^ Supported metal catalysts, especially platinum-based systems, have garnered significant attention due to their exceptional hydrogen activation capability.^7,8^ However, the catalytic performance of such systems is intricately linked not only to the active metal sites but also to the structural and electronic properties of the support material.^9–14^ Previous extensive research has predominantly focused on elucidating the internal relationship between metal nanoparticle characteristics (including size regulation, alloy composition, and morphological engineering) and the catalytic hydrogenation performance.^15–18^

The microstructures of support, particularly the exposed crystal facet, have been recognized as a critical influencing factor for catalytic hydrogenation performance by modulating the geometric/electronic configurations of metal nanoparticles,^19–22^ facilitating support-mediated reactant activation processes,^23,24^ inducing interfacial charge transfer dynamics,^25,26^ and so on. For example, Gao *et al.* reported a study on tailoring the electronic states of Pd nanoparticles by modulating the exposed crystal facets of ZIF-8 supports.^27^ By constructing sandwich-structured ZIF-8_x_@Pd@ZIF-8 composites, where distinct facets were engineered, the electron density of Pd nanoparticles was precisely controlled. The (100) facet induced electron-deficient Pd species (ZIF-8_c_@Pd@ZIF-8) could preferentially adsorb the electron-rich nitro group of *p*-chloronitrobenzene, achieving excellent catalytic activity and selectivity in the hydrogenation of *p*-chloronitrobenzene to *p*-chloroaniline. Moreover, the facet engineering of nanocatalysts has proven critical for optimizing hydrogen spillover processes for selective hydrogenation. Jiang *et al.* reported a groundbreaking study on tailoring the catalytic performance of highly diluted Pd single-atom catalysts through facet engineering of Cu supports.^28^ By dispersing Pd atoms onto Cu nanosheets (exposing (111) facets) and nanocubes (exposing (100) facets), the authors revealed a striking facet-dependent behavior in the semi-hydrogenation of alkynes. While hydrogen spillover occurred on both facets, only Pd_1_/Cu(100) exhibited exceptional activity and selectivity even at ultralow Pd loadings (50 ppm). Despite advances in catalyst design, the role of the support facet in modulating electronic states and hydrogen spillover efficiency remains poorly understood.

Herein, we systematically investigate the facet-dependent behavior of Co_3_O_4_-supported Pt catalysts. Three distinct Co_3_O_4_ morphologies—octahedral (exposing (111) facets), cubic (exposing (100) facets), and truncated intermediates (co-exposing (111) and (100) facets)—were synthesized to anchor Pt nanoparticles with similar sizes and loadings. For the 4-nitrophenol (4-NP) hydrogenation with H_2_ as the hydrogen source, the Pt/Co_3_O_4_-x (x = o, t, c) catalysts present a notable dependence of catalytic activity on exposed facets of supports. And the catalytic activity follows the order: Pt/Co_3_O_4_-o > Pt/Co_3_O_4_-t > Pt/Co_3_O_4_-c. Detailed analyses indicate that, the Pt/Co_3_O_4_-o with more Pt^0^ species has stronger hydrogen activation and spillover capacity and thus significantly accelerates the catalytic hydrogenation performance.

## Results and discussion

### Catalyst characterization

**Fig. 1:**
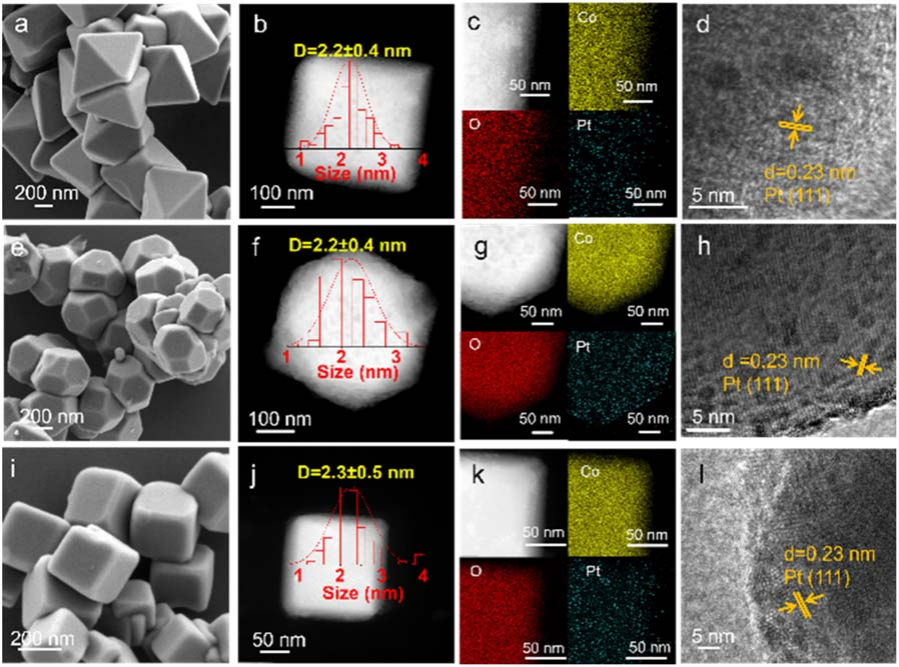
(a, e, and i) SEM images, (b, f and j) HAADF-STEM images, (c, g, and k) STEM-EDS elemental mappings, and (d, h, and l) HRTEM images of Pt/Co_3_O_4_-o, Pt/Co_3_O_4_-t, and Pt/Co_3_O_4_-c, respectively.

A series of Co_3_O_4_ supports with different morphologies were synthesized by hydrothermal method (Fig. S1). Their scanning electron microscopy (SEM) images demonstrate that the synthesized nanoparticles exhibit good morphology (Fig. S2a–c). For different morphologies of Co_3_O_4_, the corresponding exposed crystal facets are distinct. Specifically, the octahedron Co_3_O_4_ (Co_3_O_4_-o) and cube Co_3_O_4_ (Co_3_O_4_-c) are enclosed with (111) and (100) facets, respectively, while the truncated octahedron Co_3_O_4_ (Co_3_O_4_-t) exposes mixed (111) and (100) facets (Fig. S2d–f). Subsequently, the pre-synthesized Pt nanoparticles were supported on the Co_3_O_4_-x (x = o, t, c) supports *via* the colloidal deposition method with ∼0.3 wt% Pt loading (determined by ICP-OES, Table S1), yielding Pt/Co_3_O_4_-o, Pt/Co_3_O_4_-t, and Pt/Co_3_O_4_-c. SEM images of Pt/Co_3_O_4_-x (x = o, t, c) show that the size and morphology of the original Co_3_O_4_-x (x = o, t, c) are almost maintained after Pt loading (Fig. 1a, e, and i).

The high-angle annular dark field scanning transmission electron microscopy (HAADF-STEM) with energy dispersive X-ray spectroscopy (EDS) elemental mapping was further employed to characterize Pt/Co_3_O_4_-x (x = o, t, c). As shown in Fig. 1b, f, and j, obvious bright spots of Pt nanoparticles are observed. Pt nanoparticles are uniformly dispersed on the Co_3_O_4_-x (x = o, t, c) supports (Fig. 1c, g, and k). And the average diameters of Pt nanoparticles are similar, about 2.2 nm. Furthermore, the lattice fringes measured in the high-resolution TEM (HRTEM) images of Pt/Co_3_O_4_-x (x = o, t, c) (Fig. 1d, h and l) are 0.23 nm, corresponding to the (111) planes of Pt. N_2_ adsorption–desorption experiments indicate that the Brunauer–Emmett–Teller (BET) surface areas of Pt/Co_3_O_4_-o, Pt/Co_3_O_4_-t, and Pt/Co_3_O_4_-c are 2.4, 3.4, and 3.4 m^2^ g^−1^, respectively, indicating similar surface areas (Table S1).

X-ray diffraction (XRD) was used to investigate the crystal structures of Co_3_O_4_-x and Pt/Co_3_O_4_-x (x = o, t, c), as shown in Fig. S3a. All XRD patterns show the diffraction peaks located at 19.0°, 31.3°, 36.9°, 38.6°, 44.9°, 55.7°, 59.4°, and 65.3°, which correspond to the (111), (220), (311), (222), (400), (422), (511), and (440) planes of Co_3_O_4_, respectively (PDF#43-1003). The intensity ratio of the (111) to (400) peaks for Co_3_O_4_-x (x = o, t, c) decreases in order of octahedron (0.9) > truncated octahedron (0.7) > cube (0.5) (Fig. S3b), suggesting morphology-dependent facet exposure. Compared to bare Co_3_O_4_-x (x = o, t, c) supports, the Pt-loaded counterparts (Pt/Co_3_O_4_-x) exhibit similar trends in the (111)/(400) peak intensity ratios (Fig. S3c), implying that Pt deposition does not significantly perturb the predominant facet distribution of the Co_3_O_4_ supports. A semi-quantitative analysis based on the XRD intensity ratios estimates the relative abundance of the (111) facet to be ∼100%, ∼57%, and ∼0% for Pt/Co_3_O_4_-o, -t, and -c, respectively, providing a quantitative structural descriptor for the catalyst series (Table S2). No characteristic diffraction peaks belonging to crystalline Pt nanoparticles are observed, which can be attributed to the low loading and/or small size of Pt species.

**Fig. 2:**
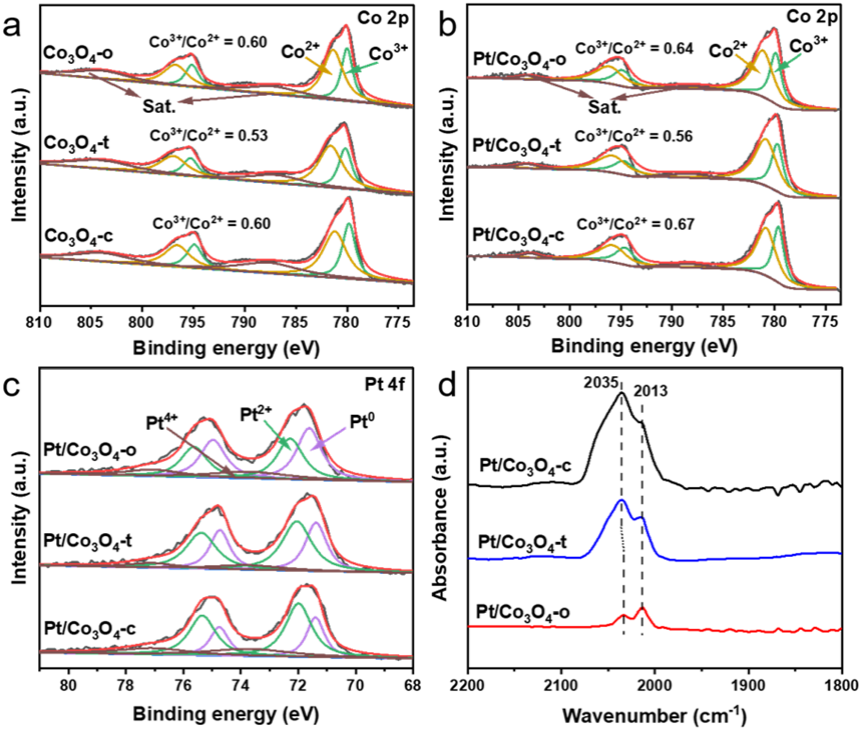
XPS spectra of Co 2p for (a) Co_3_O_4_-x and (b) Pt/Co_3_O_4_-x (x = o, t, c). (c) XPS spectra of Pt 4f and (d) CO-DRIFTS spectra for Pt/Co_3_O_4_-x (x = o, t, c).

X-ray photoelectron spectroscopy (XPS) was employed to reveal the surface chemical states of Pt and Co. Fig. 2a and b show the Co 2p spectra of Co_3_O_4_-x and Pt/Co_3_O_4_-x (x = o, t, c), respectively. The peaks at ∼780.2 and 795.4 eV are assigned to Co 2p_3/2_ and Co 2p_1/2_, respectively. The peaks of Co 2p_3/2_ can be deconvoluted into two peaks at ∼780.0 and 781.4 eV, indicating the coexistence of Co^3+^ and Co^2+^.^29^ Notably, compared to the pristine Co_3_O_4_-x (x = o, t, c) supports, the Pt-loaded counterparts (Pt/Co_3_O_4_-x) with the same morphologies show an increase in the Co^3+^/Co^2+^ ratio, implying the transfer of electrons from Co to Pt.^30^ Moreover, the XPS spectra of Pt 4f for Pt/Co_3_O_4_-x (x = o, t, c) show two peaks located at ∼71.7 and 75.0 eV, which are attributed to Pt 4f_7/2_ and 4f_5/2_, respectively (Fig. 2c). And the Pt 4f spectra were fitted by Pt^0^, Pt^2+^, and Pt^4+^.^31^ The fitting results reveal that the Pt^0^ content follows the order: Pt/Co_3_O_4_-o (44.7%) > Pt/Co_3_O_4_-t (39.4%) > Pt/Co_3_O_4_-c (26.1%) (Table S3), suggesting the differentiated electronic states of Pt nanoparticles with varied Co_3_O_4_ facets. To gain insight into the state of Pt under reaction conditions, quasi-in situ XPS analysis was performed after H_2_ treatment at 40 °C. Intriguingly, although the Pt^0^ content increased for all catalysts, the facet-dependent order remained unchanged (Fig. S4, Table S3), suggesting that the intrinsic electronic modulation by the support facet is preserved under a reducing atmosphere.

Further, diffuse-reflectance infrared Fourier transform spectroscopy (DRIFTS) of CO chemisorption was performed to evaluate the electronic states of surface Pt species in Pt/Co_3_O_4_-x (x = o, t, c), as shown in Fig. 2d. Two absorption bands observed at 2033–2035 and 2013 cm^−1^ are assigned to the linear absorption of CO on the terrace and step sites of Pt nanoparticles, respectively.^32^ Compared to the Pt/Co_3_O_4_-t and Pt/Co_3_O_4_-c, the CO adsorption peak at the terrace sites of Pt/Co_3_O_4_-o shifts to a lower wavenumber (from 2035 to 2033 cm^−1^). Additionally, the relative intensity of the CO signal adsorbed at the step sites follows the order: Pt/Co_3_O_4_-o > Pt/Co_3_O_4_-t > Pt/Co_3_O_4_-c. These results suggest that the Pt species on Pt/Co_3_O_4_-o surfaces are in a lower valence state,^33^ which aligns with the XPS result. The observed charge transfer and modified electronic state of Pt (Fig. 2) confirm that a facet-dependent metal-support interaction is effectively established through our colloidal deposition process, even in the absence of high-temperature treatment.

**Fig. 3:**
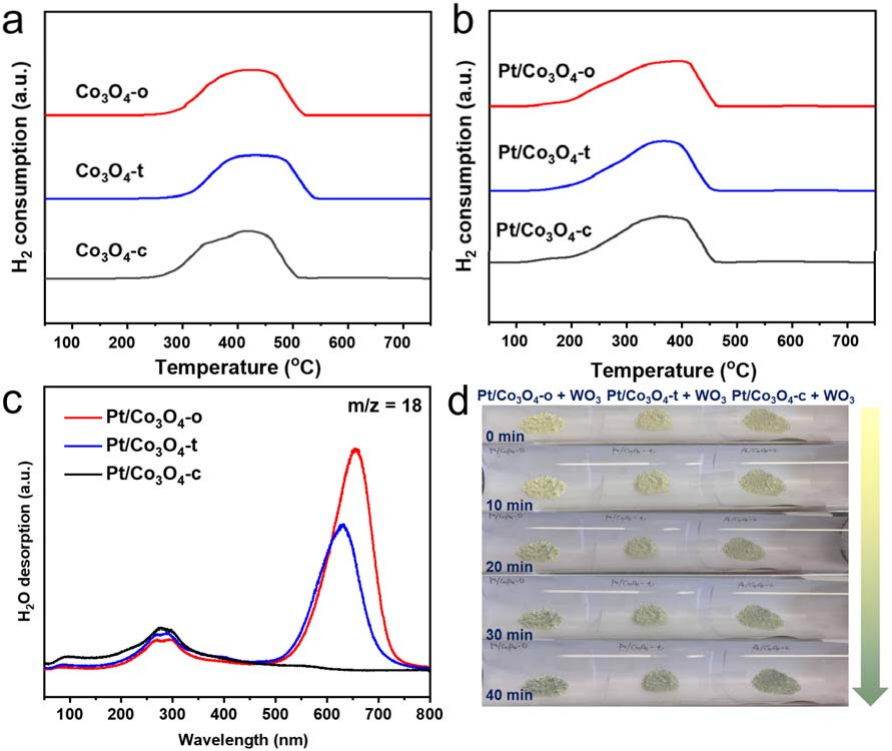
H_2_-TPR profiles of (a) the as-prepared Co_3_O_4_-x and (b) Pt/Co_3_O_4_-x (x = o, t, c) catalysts. (c) H_2_-TPD profiles of the as-prepared Pt/Co_3_O_4_-x (x = o, t, c). (d) Photographs of samples made with WO_3_ (300 mg) mixed with the Pt/Co_3_O_4_-x (x = o, t, c) catalysts (1 mg) before treatment and after treatment with 10% H_2_/Ar at 25 °C for different times.

Hydrogen temperature-programmed reduction (H_2_-TPR) was carried out to investigate the reducibility of Co_3_O_4_-x and Pt/Co_3_O_4_-x (x = o, t, c), as shown in Fig. 3a and b. H_2_-TPR profiles show no reduction peak below 100 °C for any Pt/Co_3_O_4_-x catalyst, confirming the metallic state (Pt^0^) of the pre-synthesized nanoparticles. The prominent peaks observed at higher temperatures (∼370 °C) are therefore attributed solely to the reduction of the Co_3_O_4_ support. The shift to lower temperatures (by ∼50 °C) compared to bare Co_3_O_4_ demonstrates that metallic Pt^0^ promotes support reduction *via* hydrogen spillover.^34,35^

To detect spilled hydrogen, hydrogen temperature-programmed desorption coupled with mass spectrometry (H_2_-TPD-MS) measurements of Pt/Co_3_O_4_-x were performed. As shown in Fig. 3c and S5, the MS signals reveal that the major desorption products are H_2_O (*m*/*z* = 18) and OH species (*m*/*z* = 17), with no significant molecular H_2_ (*m*/*z* = 2) detected. This indicates that active hydrogen species generated on Pt^0^ sites spill over onto the Co_3_O_4_ support and react with lattice oxygen to form surface hydroxyl groups, which subsequently recombine and decompose upon heating.^36,37^ The profiles show two main desorption regions: a peak at ∼280 °C attributed to the removal of weakly-bound hydroxyls, and another more intense peak at ∼650 °C associated with strongly-bound hydroxyls likely formed from hydrogen that has migrated deeper into the oxide lattice. Critically, the intensity of the high-temperature peak follows the order: Pt/Co_3_O_4_-o > Pt/Co_3_O_4_-t > Pt/Co_3_O_4_-c. This provides direct evidence that the (111) facet of Co_3_O_4_ is the most effective in stabilizing and incorporating spilled hydrogen.

Further, a color change experiment was conducted to visually evaluate the hydrogen spillover effect (Fig. 3d). By mixing 1 mg of Pt/Co_3_O_4_-x catalysts and 300 mg of WO_3_ nanowires, the original color of the mixtures is light yellow. After being exposed to hydrogen atmosphere, the mixture of Pt/Co_3_O_4_-o and WO_3_ exhibits the most pronounced color change within 40 minutes, implying the strongest hydrogen spillover effect over Pt/Co_3_O_4_-o. This result aligns with the H_2_-TPD-MS data and is attributed to the stronger metal-support interaction in Pt/Co_3_O_4_-o, which facilitates the generation and transfer of active hydrogen species (Fig. 2c and d and S6). Further, a parallel experiment conducted in aqueous reaction conditions (Fig. S7) also yielded a distinct color change, confirming that hydrogen spillover persists in the liquid environment relevant to catalysis.

### Catalytic performance of Pt/Co_3_O_4_-x (x = o, t, c) for 4-NP hydrogenation

**Fig. 4:**
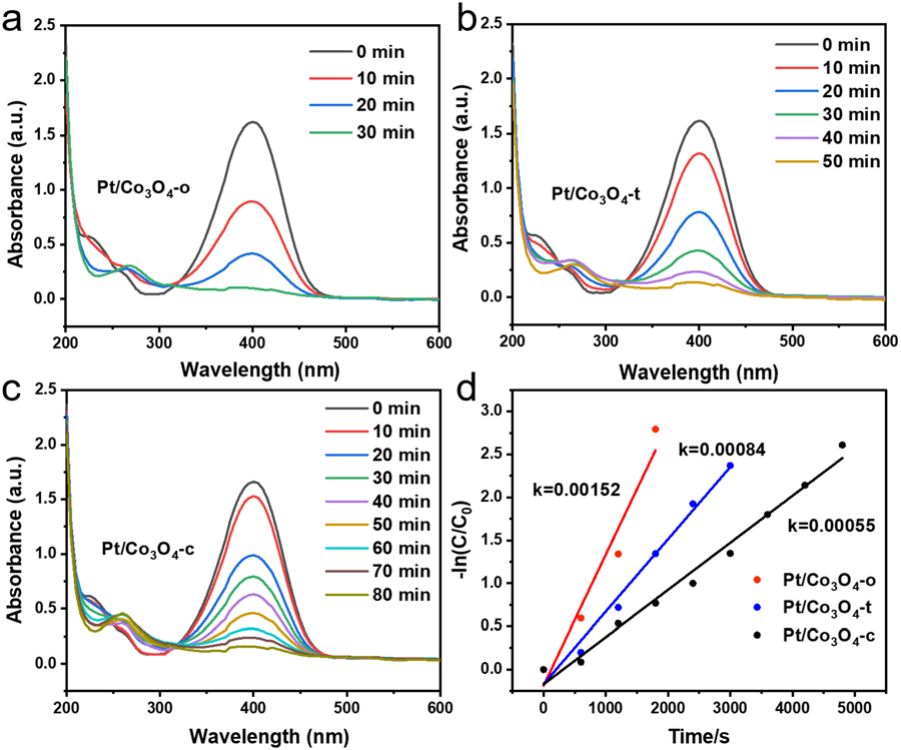
Time-dependent UV-vis spectra of 4-NP reduced by (a) Pt/Co_3_O_4_-o, (b) Pt/Co_3_O_4_-t, and (c) Pt/Co_3_O_4_-c. (d) The kinetics plots of −ln(*C*/*C*_0_) against the reaction time for the reduction of 4-NP over different Pt/Co_3_O_4_-x (x = o, t, c) catalysts.

The catalytic performance of Pt/Co_3_O_4_-x (x = o, t, c) was evaluated by the 4-NP reduction. The reduction process using H_2_ as hydrogen source was monitored by UV-vis spectroscopy every ten minutes, and the intensity of the UV absorption peak at 400 nm was used to quantify the concentration of 4-NP. As shown in Fig. 4a–c, the peak intensity at 400 nm gradually decreased as the reaction proceeded. Simultaneously, a new peak appeared at 257 nm, which was ascribed to the formation of 4-aminobenzenol (4-AP) (Fig. S8). Among the Pt/Co_3_O_4_-o, Pt/Co_3_O_4_-t, and Pt/Co_3_O_4_-c catalysts, the Pt/Co_3_O_4_-o exhibited the shortest reaction time with nearly 100% conversion (Fig. S9). Furthermore, Fig. 4d shows a logarithmic plot of the concentration (−ln(*C*/*C*_0_) *versus* reaction time for different catalysts. The concentration (−ln(*C*/*C*_0_) is proportional to the reaction time, and thus the apparent kinetic rate constant (*k*_app_) can be estimated based on slope regression of the logarithmic graph (ln(*C*_t_/*C*_0_) = −*k*_app_t).^38^ Compared to the Pt/Co_3_O_4_-t and Pt/Co_3_O_4_-c catalysts (8.4 × 10^−4^ and 5.5 × 10^−4^ s^−1^, respectively), the Pt/Co_3_O_4_-o has the highest *k*_app_ value (1.52 × 10^−3^ s^−1^), indicating its superior catalytic activity. Under identical reaction conditions, the bare Co_3_O_4_ support exhibited negligible catalytic activity (Fig. S10).

To further evaluate the intrinsic activity per Pt site, the turnover frequency (TOF) was determined based on CO chemisorption measurements. The calculated TOF values follow the same trend: Pt/Co_3_O_4_-o (164.2 h^−1^) > Pt/Co_3_O_4_-t (115.2 h^−1^) > Pt/Co_3_O_4_-c (75.6 h^−1^) (Table S4). Subsequently, the apparent activation energy (*E*_a_) experiment was performed (Fig. S11). The *E*_a_ values follow the order: Pt/Co_3_O_4_-o (23.0 kJ mol^−1^) < Pt/Co_3_O_4_-t (25.3 kJ mol^−1^) < Pt/Co_3_O_4_-c (55.2 kJ mol^−1^). The significantly lower *E*_a_ for Pt/Co_3_O_4_-o accounts for its superior catalytic activity. More importantly, we establish a quantitative correlation between the Co_3_O_4_(111) facet exposure and the TOF (Fig. S12). This linear correlation directly demonstrates that the abundance of the (111) facet is the predominant factor governing the catalytic hydrogenation activity.

For comparison, control catalysts were also prepared *via* a conventional impregnation-chemical reduction method. While the same activity order (Pt/Co_3_O_4_-o > Pt/Co_3_O_4_-c) was preserved, the catalytic activities of the impregnation-prepared catalysts were substantially lower than their colloidal-deposition counterparts (Fig. S13). This lower activity is consistent with the larger and less uniform Pt nanoparticles formed by the impregnation method (Fig. S14), demonstrating the advantage of the colloidal deposition route for achieving high Pt dispersion on our low-surface-area Co_3_O_4_ supports.

The Pt/Co_3_O_4_-o was reused to test the stability for 4-NP hydrogenation. The catalytic activity was well maintained after five cycles (Fig. S15), indicating its high catalytic stability. Post-reaction characterizations (XRD, TEM, and XPS) confirm that the Pt nanoparticle size, dispersion, and chemical state, as well as the Co_3_O_4_ crystal facets, remain essentially unchanged after cycling (Fig. S16 and Table S3). Together with the minimal Pt leaching (<0.2%) confirmed by ICP-MS (Table S5), these results demonstrate the robust structural and compositional stability of the catalyst. When nitrobenzene was employed as the substrate, the Pt/Co_3_O_4_-o still exhibited the superior catalytic activity (Fig. S17 and S18).

Compared with state-of-the-art catalysts for 4-NP hydrogenation (Table S6), the optimal Pt/Co_3_O_4_-o demonstrates a competitive turnover frequency (164.2 h^−1^) under an exceptionally mild condition (40 °C, 1 bar H_2_). Many reported systems require significantly higher H_2_ pressures or temperatures to achieve comparable activity. This combination of high intrinsic activity, low activation barrier, robust stability, and facet-dependent performance underscores the practical promise and fundamental insight offered by our facet-engineered catalyst.

### Catalytic mechanism

**Fig. 5:**
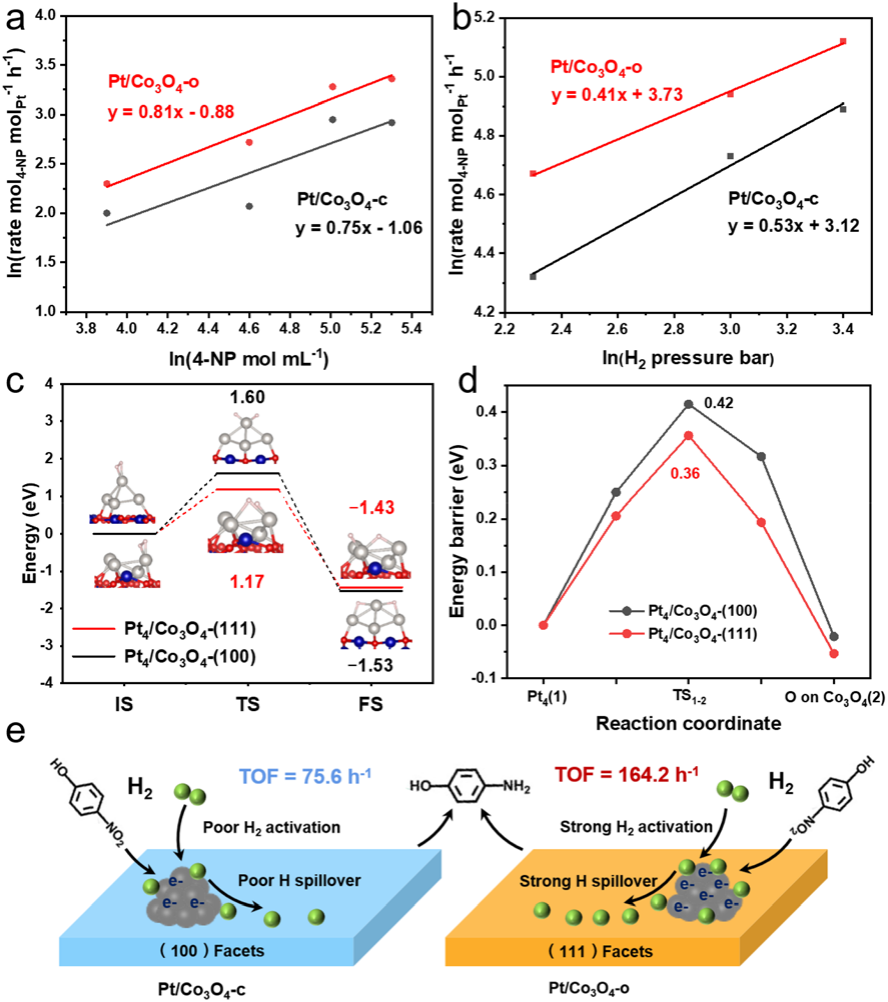
Kinetic experiments of 4-NP hydrogenation over the Pt/Co_3_O_4_-o and Pt/Co_3_O_4_-c catalysts: the reaction order for (a) 4-NP and (b) H_2_. The reaction rates were determined at conversions below 30%. (c) Potential energy profiles of H_2_ dissociation on the Pt_4_/Co_3_O_4_-(100) and Pt_4_/Co_3_O_4_-(111) model surfaces. (d) Potential energy profiles for hydrogen atom migration from Pt_4_ to Co_3_O_4_ across the interface on the Pt_4_/Co_3_O_4_-(100) and Pt_4_/Co_3_O_4_-(111) model surfaces. (e) Schematic illustration of possible mechanisms on Pt/Co_3_O_4_-c and Pt/Co_3_O_4_-o for 4-NP hydrogenation.

To elucidate the origin of the superior catalytic performance of Pt/Co_3_O_4_-o, diffuse reflectance infrared Fourier transform spectroscopy (DRIFTS) was first used to investigate the adsorption behavior of 4-NP on Pt/Co_3_O_4_-o and Pt/Co_3_O_4_-c as model catalysts, as shown in Fig. S19. The spectra of Pt/Co_3_O_4_-o and Pt/Co_3_O_4_-c show two bands at 1578 and 1316 cm^−1^, corresponding to asymmetric stretching and symmetric stretching vibrations of the nitro group, respectively.^39^ Compared with the Pt/Co_3_O_4_-c catalyst, Pt/Co_3_O_4_-o exhibits a weaker peak intensity at 1578 cm^−1^, suggesting a weaker adsorption capability for the nitro group. Further, the adsorption and activation capability of Pt/Co_3_O_4_-o and Pt/Co_3_O_4_-c for 4-NP and H_2_ was explored through kinetic experiments. Fig. 5a displays the kinetic behavior for 4-NP adsorption and activation. The reaction order with respect to 4-NP is higher for Pt/Co_3_O_4_-o (0.81) than for Pt/Co_3_O_4_-c (0.75), implying that it is more difficult for Pt/Co_3_O_4_-o to activate the nitro group. This is consistent with the result of DRIFTS. For the adsorption and activation of H_2_ (Fig. 5b), the reaction order of Pt/Co_3_O_4_-o (0.41) is lower than that of Pt/Co_3_O_4_-c (0.53), indicating that it is easier for Pt/Co_3_O_4_-o to activate H_2_.

To gain theoretical insight, DFT calculations were conducted. Pt_4_ clusters supported on Co_3_O_4_-(111) and Co_3_O_4_-(100) (labeled as Pt_4_/Co_3_O_4_-(111) and Pt_4_/Co_3_O_4_-(100), respectively) were constructed to mimic the Pt/Co_3_O_4_-o and Pt/Co_3_O_4_-c catalysts based on the distinct exposed facets of Co_3_O_4_ supports: the octahedral Co_3_O_4_-o predominantly exposes the (111) facet, whereas the cubic Co_3_O_4_-c preferentially exposes the (100) facet (Fig. S20a and b). First, when Pt_4_ clusters were anchored on different Co_3_O_4_ model surfaces, the electron transfer from Co_3_O_4_ to Pt_4_ was about −1.65|*e*| for Pt_4_/Co_3_O_4_-(111) and −0.74|*e*| for Pt_4_/Co_3_O_4_-(100), respectively (Fig. S20c and d, Table S7). The charge density differences indicate that more electrons transfer from Co_3_O_4_ to Pt for Pt_4_/Co_3_O_4_-(111), leading to a lower oxidation state of Pt. This is also confirmed by the XPS results. Next, Fig. S21a–d shows the optimized adsorption structures of 4-NP on the Pt_4_/Co_3_O_4_-(111) and Pt_4_/Co_3_O_4_-(100) surfaces. The adsorption energy results presented in Fig. S21e indicate that the Pt_4_/Co_3_O_4_-(111) exhibits the weaker capability for facilitating the nitro group activation. Further, Fig. 5c reveals the adsorption and activation of H_2_ on the Pt_4_/Co_3_O_4_-(111) and Pt_4_/Co_3_O_4_-(100) surfaces, in which the energy barrier (1.17 eV) of H_2_ dissociation on Pt_4_/Co_3_O_4_-(111) is lower than that on Pt_4_/Co_3_O_4_-(100) (1.6 eV), implying that the activation of H_2_ by Pt_4_/Co_3_O_4_-(111) is easier. These results are consistent with the kinetics results. Furthermore, for hydrogen-atom migration from Pt to Co_3_O_4_ across the interface, the energy barrier on the Pt_4_/Co_3_O_4_-(111) (0.36 eV) is lower than that on the Pt_4_/Co_3_O_4_-(100) (0.42 eV), indicating kinetically more favourable hydrogen spillover on Co_3_O_4_-(111) (Fig. 5d and S22).

Based on the above results, the possible promotion mechanism is proposed (Fig. 5e). For the hydrogenation reaction, the activity of a catalyst is strongly related to the substrate activation ability, H_2_ activation ability, as well as active hydrogen spillover.^40,41^ With Pt/Co_3_O_4_-o and Pt/Co_3_O_4_-c as model catalysts, the kinetic experiments and DFT calculations illustrate that compared with Pt/Co_3_O_4_-c, the adsorption/activation of 4-NP on the Pt/Co_3_O_4_-o is weaker, indicating that the adsorption/activation of 4-NP is not the key factor contributing to the difference in activity in this reaction. However, the Pt/Co_3_O_4_-o has a stronger H_2_ dissociation ability and lower hydrogen-migration barrier. The accelerated hydrogen dissociation on electron-rich Pt sites, coupled with enhanced hydrogen spillover to the Co_3_O_4_ support, establishes a dynamic hydrogen supply chain that maximizes the utilization of active hydrogen species. The 4-NP adsorbed on Pt or near Pt-Co_3_O_4_ interfaces can continuously consume active hydrogen species, and the hydrogenation reaction can be promoted in a dynamic process.

## Conclusions

In summary, a series of well-defined Pt/Co_3_O_4_-x (x = o, t, c) catalysts with tailored Co_3_O_4_ crystal facets have been successfully constructed for the hydrogenation of 4-nitrophenol. Owing to the crystal facet effect, the Pt nanoparticles in Pt/Co_3_O_4_-o are more negatively charged than those in Pt/Co_3_O_4_-c and Pt/Co_3_O_4_-t, and Pt/Co_3_O_4_-o exhibits the strongest hydrogen spillover capability. For the 4-nitrophenol (4-NP) hydrogenation reaction, the Pt/Co_3_O_4_-o has the best catalytic activity with a TOF value of 164.2 h^−1^. Detailed analyses reveal that the negatively charged Pt sites are favorable to the activation of hydrogen rather than the nitro group. Therefore, the enhanced catalytic activity of Pt/Co_3_O_4_-o is attributed to the promoted hydrogen activation and spillover. This work highlights crystal facet engineering of support to regulate the electronic structure and hydrogen spillover effect, which provides in-depth insight into the catalyst design and hydrogenation mechanism.

## Author contributions

The manuscript was written through contributions of all authors. All authors have given approval to the final version of the manuscript. Hui Yun: investigation, validation, visualization, data curation, formal analysis, methodology, writing – original draft. Jiao Feng: visualization, writing – review & editing. Wanying Peng: visualization, writing – review & editing. Mi Xiong: conceptualization, funding acquisition, supervision, writing – review & editing.

## Conflicts of interest

There are no conflicts to declare.

## Supplementary Material

SC-OLF-D5SC09402J-s001

## Data Availability

The data supporting this article has been included as part of the supplementary information (SI). Supplementary information is available. See DOI: https://doi.org/10.1039/d5sc09402j.

## References

[cit1] Wang D., Astruc D. (2015). The Golden Age of Transfer Hydrogenation. Chem. Rev..

[cit2] Karim A. V., Cichocki Ł., Wang C., Boczkaj G. (2025). Advanced reduction processes (ARPs) based on catalytic hydrogenation for wastewater pollutants degradation - a special focus on process efficiency and mechanisms – a review. J. Cleaner Prod..

[cit3] Luo S., Liu Z., Liu Y., Almatrafi E., Shao B., Song B., Zhou C., Fu Y., He M., Zeng Z., Zeng G. (2022). Versatile CMPs as platforms to support Ag nanocatalysts for nitrophenol hydrogenation in continuous flow-through process. Chem. Eng. J..

[cit4] Lai B., Chen Z., Zhou Y., Yang P., Wang J., Chen Z. (2013). Removal of high concentration p-nitrophenol in aqueous solution by zero valent iron with ultrasonic irradiation (US-ZVI). J. Hazard. Mater..

[cit5] Lai B., Zhang Y. H., Li R., Zhou Y. X., Wang J. (2014). Influence of operating temperature on the reduction of high concentration p-nitrophenol (PNP) by zero valent iron (ZVI). Chem. Eng. J..

[cit6] Ren Y., Li J., Lai L., Lai B. (2017). Premagnetization enhancing the reactivity of Fe^0^/(passivated Fe^0^) system for high concentration p-nitrophenol removal in aqueous solution. Chemosphere.

[cit7] Zhang Y., Zhou J. (2021). Synergistic catalysis by a hybrid nanostructure Pt catalyst for high-efficiency selective hydrogenation of nitroarenes. J. Catal..

[cit8] Zhang Q., Bu J., Wang J., Sun C., Zhao D., Sheng G., Xie X., Sun M., Yu L. (2020). Highly Efficient Hydrogenation of Nitrobenzene to Aniline over Pt/CeO_2_ Catalysts: The Shape Effect of the Support and Key Role of Additional Ce^3+^ Sites. ACS Catal..

[cit9] Wang Y., Wang C., Wang L., Wang L., Xiao F. S. (2021). Zeolite Fixed Metal Nanoparticles: New Perspective in Catalysis. Acc. Chem. Res..

[cit10] Pacchioni G., Freund H. J. (2018). Controlling the charge state of supported nanoparticles in catalysis: lessons from model systems. Chem. Soc. Rev..

[cit11] van Deelen T. W., Hernández Mejía C., de Jong K. P. (2019). Control of metal-support interactions in heterogeneous catalysts to enhance activity and selectivity. Nat. Catal..

[cit12] Xiong M., Li Y., Sun L., Hu Q., Lv Z., Xing S., Yun H., Zhang S., Gao Z. (2025). Magnetically recyclable bimetallic Pt-Co/CoO catalyst derived through hydrogen spillover for fast hydrogen release through ammonia borane hydrolysis. Surf. Interfaces.

[cit13] Zhang P., Qin T., Li D., Wu X. Q., Ma Y. X., Guo H. Q., Xiong J., Liu X., Zhao Z., Chen L. W., Liu J., Wei Y. C. (2025). Temperature-induced evolution of CuO_x_ clusters in CuO_x_/TiO_2_ catalyst for boosting auto-exhaust oxidation. Appl. Catal. B Environ. Energy.

[cit14] Li Y. F., Qin T., Wei Y. C., Xiong J., Zhang P., Lai K. Z., Chi H. J., Liu X., Chen L. W., Yu X. L., Zhao Z., Li L. N., Liu J. (2023). A single site ruthenium catalyst for robust soot oxidation without platinum or palladium. Nat. Commun..

[cit15] Ahmadi M., Mistry H., Roldan Cuenya B. (2016). Tailoring the Catalytic Properties of Metal Nanoparticles via Support Interactions. J. Phys. Chem. Lett..

[cit16] Li Z., Ji S., Liu Y., Cao X., Tian S., Chen Y., Niu Z., Li Y. (2019). Well-Defined Materials for Heterogeneous Catalysis: From Nanoparticles to Isolated Single-Atom Sites. Chem. Rev..

[cit17] Liu L., Corma A. (2018). Metal Catalysts for Heterogeneous Catalysis: From Single Atoms to Nanoclusters and Nanoparticles. Chem. Rev..

[cit18] Liu X., Wang X., Zhen S., Sun G., Pei C., Zhao Z. J., Gong J. (2022). Support stabilized PtCu single-atom alloys for propane dehydrogenation. Chem. Sci..

[cit19] Huang X., Dang C., Yu H., Wang H., Peng F. (2015). Morphology Effect of Ir/La_2_O_2_CO_3_ Nanorods with Selectively Exposed {110} Facets in Catalytic Steam Reforming of Glycerol. ACS Catal..

[cit20] Hu Z., Zou Z., Xie A., Chen C., Zhu X., Zhang Y., Zhang H., Zhao H., Wang G. (2021). Crystal plane effect of ceria on supported copper catalyst for liquid-phase hydrogenation of unsaturated aldehyde. J. Colloid Interface Sci..

[cit21] Zhang P., Xiong J., Wei Y. C., Li Y. F., Zhang Y. L., Tang J. J., Song W. Y., Zhao Z., Liu J. (2021). Exposed {001} facet of anatase TiO_2_ nanocrystals in Ag/TiO_2_ catalysts for boosting catalytic soot combustion: The facet-dependent activity. J. Catal..

[cit22] Wang S., Liu C., Hao W., Zhuang Y., Chen J., Zhu X., Wang L., Niu X., Mao J., Ma D., Zhao Q. (2025). Structural evolution of metal single-atoms and clusters in catalysis: Which are the active sites under operative conditions?. Chem. Sci..

[cit23] Cargnello M., Doan-Nguyen V. V. T., Gordon T. R., Diaz R. E., Stach E. A., Gorte R. J., Fornasiero P., Murray C. B. (2013). Control of Metal Nanocrystal Size Reveals Metal-Support Interface Role for Ceria Catalysts. Science.

[cit24] Huang G., Yang Q. H., Xu Q., Yu S. H., Jiang H. L. (2016). Polydimethylsiloxane Coating for a Palladium/MOF Composite: Highly Improved Catalytic Performance by Surface Hydrophobization. Angew. Chem., Int. Ed..

[cit25] Fu Q., Wagner T. (2007). Interaction of nanostructured metal overlayers with oxide surfaces. Surf. Sci. Rep..

[cit26] Zhou J. F., Peng B., Ding M., Shan B. Q., Zhu Y. S., Bonneviot L., Wu P., Zhang K. (2024). The nature of crystal facet effect of TiO_2_-supported Pd/Pt catalysts on selective hydrogenation of cinnamaldehyde: electron transfer process promoted by interfacial oxygen species. Phys. Chem. Chem. Phys..

[cit27] Gao M. L., Li L., Sun Z. X., Li J. R., Jiang H. L. (2022). Facet Engineering of a Metal-Organic Framework Support Modulates the Microenvironment of Palladium Nanoparticles for Selective Hydrogenation. Angew. Chem., Int. Ed..

[cit28] Jiang L., Liu K., Hung S. F., Zhou L., Qin R., Zhang Q., Liu P., Gu L., Chen H. M., Fu G., Zheng N. (2020). Facet engineering accelerates spillover hydrogenation on highly diluted metal nanocatalysts. Nat. Nanotechnol..

[cit29] Cai Z., Bi Y., Hu E., Liu W., Dwarica N., Tian Y., Li X., Kuang Y., Li Y., Yang X. Q., Wang H., Sun X. (2017). Single-Crystalline Ultrathin Co_3_O_4_ Nanosheets with Massive Vacancy Defects for Enhanced Electrocatalysis. Adv. Energy Mater..

[cit30] Li Z., He T., Matsumura D., Miao S., Wu A., Liu L., Wu G., Chen P. (2017). Atomically Dispersed Pt on the Surface of Ni Particles: Synthesis and Catalytic Function in Hydrogen Generation from Aqueous Ammonia–Borane. ACS Catal..

[cit31] Jeong H., Shin D., Kim B. S., Bae J., Shin S., Choe C., Han J. W., Lee H. (2020). Controlling the Oxidation State of Pt Single Atoms for Maximizing Catalytic Activity. Angew. Chem., Int. Ed..

[cit32] Zhang J., Pan Y., Feng D., Cui L., Zhao S., Hu J., Wang S., Qin Y. (2023). Mechanistic insight into the synergy between platinum single atom and cluster dual active sites boosting photocatalytic hydrogen evolution. Adv. Mater..

[cit33] Zhang T., Jin J., Chen J., Fang Y., Han X., Chen J., Li Y., Wang Y., Liu J., Wang L. (2022). Pinpointing the axial ligand effect on platinum single-atom-catalyst towards efficient alkaline hydrogen evolution reaction. Nat. Commun..

[cit34] Zhang J., Chen W., Ge H., Chen C., Yan W., Gao Z., Gan J., Zhang B., Duan X., Qin Y. (2018). Synergistic effects in atomic-layer-deposited PtCox/CNTs catalysts enhancing hydrolytic dehydrogenation of ammonia borane. Appl. Catal. B Environ. Energy.

[cit35] Ouyang B., Xiong S., Zhang Y., Liu B., Li J. (2017). The study of morphology effect of Pt/Co_3_O_4_ catalysts for higher alcohol synthesis from CO_2_ hydrogenation. Appl. Catal., A.

[cit36] Li H., Abdelgaid M., Paudel J. R., Holzapfel N. P., Augustyn V., McKone J. R., Mpourmpakis G., Crumlin E. J. (2025). Operando unveiling of hydrogen spillover mechanisms on tungsten oxide surfaces. J. Am. Chem. Soc..

[cit37] Liu Y., Zhang R., Lin L., Wang Y., Liu C., Mu R., Fu Q. (2023). Direct observation of accelerating hydrogen spillover via surface-lattice-confinement effect. Nat. Commun..

[cit38] Cheng Z., He B., Zhou L. (2015). A general one-step approach for in situ decoration of MoS_2_ nanosheets with inorganic nanoparticles. J. Mater. Chem. A.

[cit39] Feng H., Yang Y., Niu Y., Wang L., Yin P., Hong S., Zhang B., Zhang X., Wei M. (2022). Highly-efficient RuNi single-atom alloy catalysts toward chemoselective hydrogenation of nitroarenes. Nat. Commun..

[cit40] Zhao Z., Li X., Liu X., Gao H., Jia A., Xie S., Song X., Liu X., Yang F., Yang Q. (2024). Pt/Fe-TiO_2_-Catalyzed Selective Carbonyl Hydrogenation: Fe-Promoted Hydrogen Spillover. ACS Catal..

[cit41] Xie L., Liang J., Jiang L., Huang W. (2025). Effects of oxygen vacancies on hydrogenation efficiency by spillover in catalysts. Chem. Sci..

