# Supplementary material for: Promoted hydrogen activation and spillover over Pt/Co3O4 by facet engineering of Co3O4 for enhanced catalytic hydrogenation
Source: Chem Sci. 2026 Mar 13;17(17):8753–9. doi: 10.1039/d5sc09402j (PMC12983403; doi:10.1039/d5sc09402j)
Supplement: SC-017-D5SC09402J-s001 [file SC-017-D5SC09402J-s001.pdf]

Supplementary Information (SI) for Chemical Science.
This journal is © The Royal Society of Chemistry 2026

Supporting Information

Promoted hydrogen activation and spillover over Pt/Co_3O_4 by facet engineering of Co_3O_4 for enhanced catalytic hydrogenation

Hui Yun, Jiao Feng, Wanying Peng, Mi Xiong*

College of Materials and Chemistry & Chemical Engineering, Chengdu University of Technology, Chengdu 610059, China
Corresponding authors: xiongmi@cdut.edu.cn

Experimental sections

Chemicals and materials

Cobalt nitrate hexahydrate ($Co(NO_3)_2{\cdot}6H_2O$, 99.99%), oxalic acid ($H_2C_2O_4$, 98%), sodium oxalate (NaC_2O_4, 99.5%), sodium hydroxide (NaOH, 95%) and 4-nitrophenol (4-NP) were purchased from Macklin. Chloroplatinic acid ($H_2PtCl_6{\cdot}6H_2O$, Pt⩾37.5%) was obtained from Aladdin. Ethylene glycol ($(CH_2OH)_2$, 99%) was purchased from Shanghai Titan Technology. Potassium hydroxide (KOH, AR) was supplied from Chengdu Kelong.

Catalyst preparation

Synthesis of Co_3O_4-x (x = o (octahedron), t (truncated octahedron), and c (cube)). Co_3O_4-x particles with different morphologies were synthesized according to the previous report with minor modifications[1].To synthesize Co_3O_4-c, 0.2 g of NaOH and 5.86 g of $Co(NO_3)_2{\cdot}6H_2O$ were dissolved in 20 mL of distilled water. The suspension was transferred to a 50 mL Teflon-lined stainless steel autoclave and then heated at 180 °C for 5 h. Co_3O_4-t was prepared by mixing 0.423 g $H_2C_2O_4$, 0.5 g NaOH, and 6.18 g $Co(NO_3)_2{\cdot}6H_2O$ in 17.5 mL of distilled water. To prepare Co_3O_4-o, 0.5 g $Na_2C_2O_4$, 0.2 g NaOH, and 6.18 g $Co(NO_3)_2{\cdot}6H_2O$ were added to 17.5 mL of distilled water. The two mixtures were then transferred to an autoclave where they were heated at 220 °C for 20 h. Following the cooling of the autoclave to ambient temperature, the respective sediments were collected separately and washed several times with distilled water and ethanol. Subsequently, the resultant products were dried at 60 °C for 5 h and then calcined in air at 500 °C for 3 h.

Synthesis of Pt NPs. Colloidal Pt NPs were prepared according to the previous report with minor modifications[2]. Specifically, 100 mg of H_2PtCl_6 was dissolved within 18 mL of $(CH_2OH)_2$, following by the addition of 144 mg of NaOH. The resulting mixture was gradually heated to 160 °C and maintained at this temperature for 3 h to obtain a black solution of colloidal Pt NPs. Following the cooling to room temperature, the colloidal Pt NPs solution was stored in a glass container.

Synthesis of Pt/Co_3O_4-x (x = o, t, c). Typically, 100 mg of Co_3O_4-c was dispersed in 10 mL of distilled water, and then 200 μL of Pt NPs colloidal solution

was added dropwise to the mixture with vigorous stirring. Next, the mixture was stirred at 25 ^{o}C for 8 h to ensure effective chemical adsorption and anchoring of the Pt nanoparticles onto the Co_3O_4 support surfaces. The Pt/Co_3O_4-c catalyst was obtained by repeated centrifugation, washing with deionized water, and drying under vacuum. The synthesis methods of Pt/Co_3O_4-t and Pt/Co_3O_4-o were analogous to that of Pt/Co_3O_4-c. Notably, no post-deposition annealing or in-situ reduction was performed to the fresh catalysts prior to reaction and characterization except for CO-pulse chemisorption.

Synthesis of Pt/Co_3O_4-x(Im) via impregnation-chemical reduction. Typically, 200 mg of Co_3O_4-x (x = o or c) support was dispersed in 10 mL of deionized water, followed by the slow addition of 2.12 mL of an aqueous H_2PtCl_6 solution (2.5 mg/mL) to the dispersion. The mixture was magnetically stirred, and then 20 mL of a freshly prepared $NaBH_4$ aqueous solution (0.242 mg/mL) was introduced to reduce the Pt precursor. After continuous stirring for 30 minutes, the solid product was washed repeatedly with ethanol and deionized water, and then vacuum-dried for 6 h to obtain the target catalyst.

Catalyst characterizations

Scanning electron microscopy (SEM) images were taken with Zeiss Sigma 360. High-resolution transmission electron microscopy (HRTEM), high-angle annular dark-field scanning transmission electron microscopy (HAADF-STEM), and energy dispersive X-ray spectroscopy (EDX) images were taken with a JEOL JEM F200 (Japan) apparatus. X-ray diffraction (XRD) patterns were acquired using a Rigaku Smart Lab SE (Japan) X-ray diffractometer equipped with Cu Kα radiation, with data collected over a 2θ range of 5° to 90° at a scanning rate of $5°\cdot min^{-1}$. The specific surface areas of all catalysts were determined via the Brunauer-Emmett-Teller (BET) method using a PM2-1568 analyzer (Beijing Beishide). The Pt loading was determined by inductively coupled plasma optical emission spectrometry (ICP-OES, Agilent 5800). Quasi-in situ XPS measurements were conducted to probe the catalyst's electronic state under a reactive H_2 atmosphere. A pressed powder sample was first analyzed to obtain the "fresh-state" spectra. It was then subjected to in situ reduction inside a dedicated reaction cell (40 °C, 50 mL min^{-1} H_2, 30 min) connected to the XPS system. Finally, the sample was transferred under vacuum back to the analysis chamber for XPS measurement without air exposure, all while maintaining the same analysis position to ensure direct comparability.

For CO-pulse chemisorption, the sample was first reduced in a 10% H_2/Ar flow by heating to 200 °C at a ramp rate of 10 °C min^{-1} and holding for 1 h. After cooling to 50 °C, the sample was purged with He for 30 min to remove physiosorbed and gaseous hydrogen. Then, pulses of 10% CO/He were injected at regular intervals until saturation adsorption was achieved.

CO chemisorption and 4-NP chemisorption measurements were performed on a Bruker INVENIO-S Fourier-transform infrared (FTIR) spectrometer (Harrick, Germany). For CO chemisorption, the sample was initially pretreated in Ar at 150 ^{o}C for 1 h to remove the moisture. After cooling the sample to 30 ^{o}C under Ar, CO was introduced until the adsorption saturation. Finally, the sample was purged by Ar until

the spectrum remained unchanged, and the spectrum was then recorded. For 4-NP chemisorption, 20 mg of sample was firstly premixed with the 4-NP solution, and then placed into the sample cell. The sample was purged with Ar at 40 °C for 1 h, and then the spectrum was recorded.

Hydrogen temperature-programmed reduction (H_2-TPR) was performed on a TP-5080 (China) instrument. 50 mg of sample was first pretreated under He (50 mL·min^{-1}) at 150 °C for 1 h to remove the moisture. After cooling to 50 °C, 10% H_2/Ar (50 mL·min^{-1}) was introduced into the system and the sample was reduced from room temperature to 800 °C with a heating rate of 10 °C·min^{-1}. Hydrogen temperature-programmed desorption mass spectrometry (H_2-TPD-MS) was performed on a TP-5080 instrument equipped with an online mass spectrometer (MS). Typically, 50 mg of sample was first pretreated under a He flow (50 mL·min^{-1}) at 150 °C for 1 h to remove moisture and adsorbed impurities. After cooling to 50 °C in He, the gas was switched to 10% H_2/Ar (30-50 mL·min^{-1}) and maintained until adsorption saturation. Subsequently, the system was purged with an Ar flow (30-50 mL·min^{-1}) for 1 h to remove physically adsorbed H_2. Finally, temperature-programmed desorption was carried out by heating the sample from room temperature to 800 °C at a rate of 10 °C·min^{-1} under an Ar atmosphere. The desorbed gases were continuously analyzed by the online mass spectrometer, with the signals for H_2 (m/z = 2), H_2O (m/z = 18), and OH-related fragments (m/z = 17) being specifically monitored.

Catalytic tests

Hydrogenation of 4-NP was selected as a model reaction to study the catalytic performance of these catalysts. The experiment was carried out in a 100 mL three-necked round bottom flask fitted with a magnetic stirrer in a water bath. In a typical process, 20 mg of catalyst was dispersed in 50 mL of 4-NP aqueous solution (0.1 mM) containing 5 mg KOH, which was added to deprotonate 4-NP to 4-nitrophenolate, shifting its characteristic UV-vis absorption peak to 400 nm for unambiguous kinetic monitoring (Figure S8). And the reaction was conducted in a hydrogen atmosphere at atmospheric pressure with magnetic stirring (900 r·min^{-1}) at 40 °C. The reaction temperature provides a practically useful reaction rate under ambient H_2 pressure, ensures clear kinetic differentiation among catalysts, and avoids side reactions that could obscure intrinsic facet-dependent performance. The reaction solution was collected every 10 minutes and detected by UV-vis spectroscopy. For the hydrogenation of nitrobenzene (NP), the process was similar to the above description with the exception that the NP aqueous solution (0.01 mM) was used without KOH (Figure S17).

The reusability of Pt/Co_3O_4-o for the hydrogenation of 4-nitrophenol was conducted under the same conditions as described above. After each cycle, the catalyst was recovered by centrifugation, washed, dried, and then reused. The cyclic process was repeated for a total of five times. Following the fifth cycle, the reaction mixture was centrifuged to separate the catalyst, and the clear supernatant was analyzed by inductively coupled plasma mass spectrometry (ICP-MS) to quantify any leached Pt species. The measured Pt concentration was used to calculate the leaching rate relative to the total Pt loading.

Calculation methods

All DFT calculations were implemented in the Vienna Ab initio Computational Simulation Package (VASP)[3]. The interactions between ionic nuclei and valence electrons were treated by the projection-enhanced wave (PAW) method[4]. The exchange-correlation potential was described by the generalized gradient approximation of Perdew-Burke-Ernzerhof (GGA-PBE)[5]. To better describe the strongly correlated electrons, a Hubbard U correction (Ueff = 3.0 eV) was applied to the 3*d* orbitals of Co and Pt atoms[6]. A plane-wave kinetic energy cutoff of 400 eV was used. For the calculation of hydrogen migration barriers, the Brillouin zone was sampled with a 3×3×1 Monkhorst-Pack *k*-point grid[7]. The convergence criteria for forces and energies during structural relaxation were -0.02 eV/Å and 10^{-4} eV, respectively[8]. Dispersive interactions between all atoms in the adsorption model were described by grime's DFT-D3 method. In order to prevent interactions between the two periodic units, a vacuum layer of 14 Å in the Z direction was used.

The adsorption energy was calculated using the formula:

$$E_{ads} = E_{Pt4/Co3O4-C6H5NO3} - E_{Pt4/Co3O4} - E_{C6H5NO3}$$

where $E_{Pt4/Co3O4-C6H5NO3}$, $E_{Pt4/Co3O4}$ and $E_{C6H5NO3}$ represent the energy of Pt_4/Co_3O_4 after adsorption of $C_6H_5NO_3$, the energy of Pt_4/Co_3O_4 and the energy of $C_6H_5NO_3$, respectively.

The minimum energy paths and the associated energy barriers for hydrogen atom migration from Pt clusters to the Co_3O_4 support were determined using the climbing-image nudged elastic band (CI-NEB) method[9].

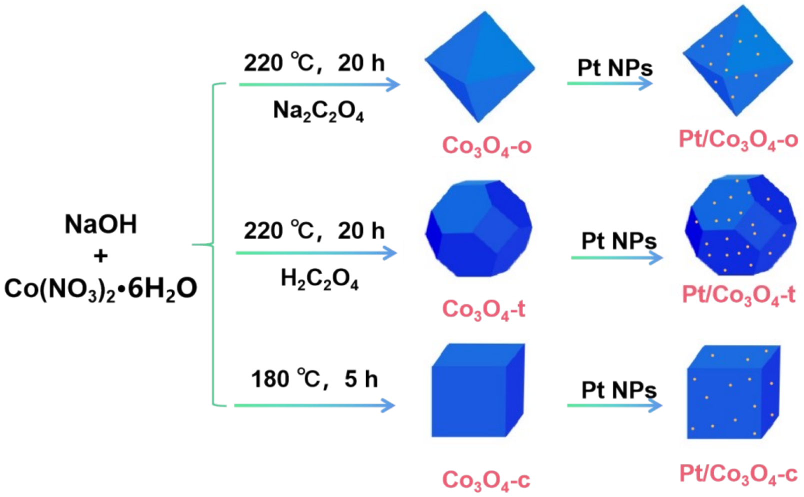

Figure S1. Schematic illustration for the preparation of Co_3O_4-x and Pt/Co_3O_4-x (x =o, t, c).

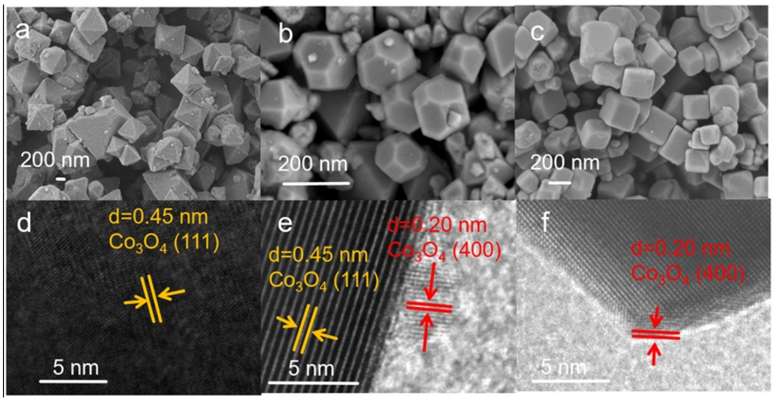

Figure S2. SEM and HRTEM images of (a, d) Co_3O_4-o, (b, e) Co_3O_4-t, and (c, f) Co_3O_4-c.

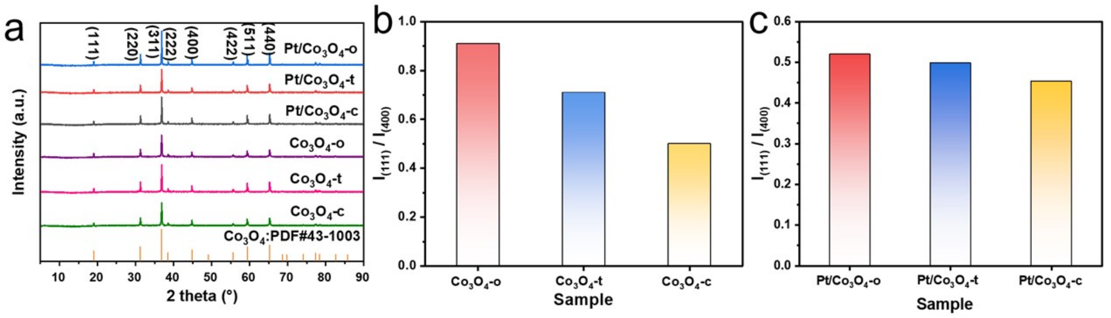

Figure S3. (a) XRD patterns of Co_3O_4-x and Pt/Co_3O_4-x (x = o, t, c). (b-c) The ratio of (111) to (400) calculated from the peak intensity in XRD pattern of (b) Co_3O_4-x and (c) Pt/Co_3O_4-x (x = o, t, c).

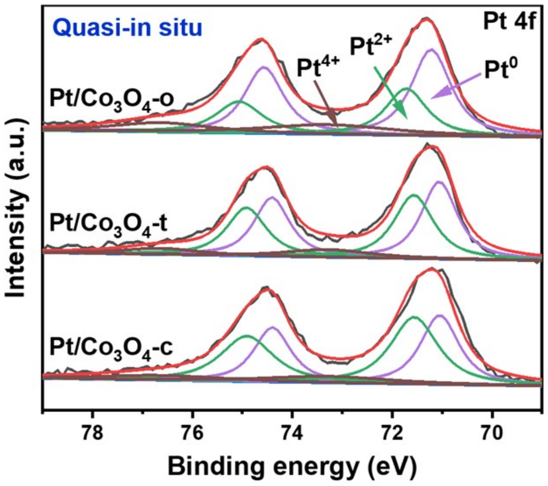

Figure S4. Quasi-in situ XPS spectra of Pt 4f for Pt/Co_3O_4-x (x = o, t, c) catalysts.

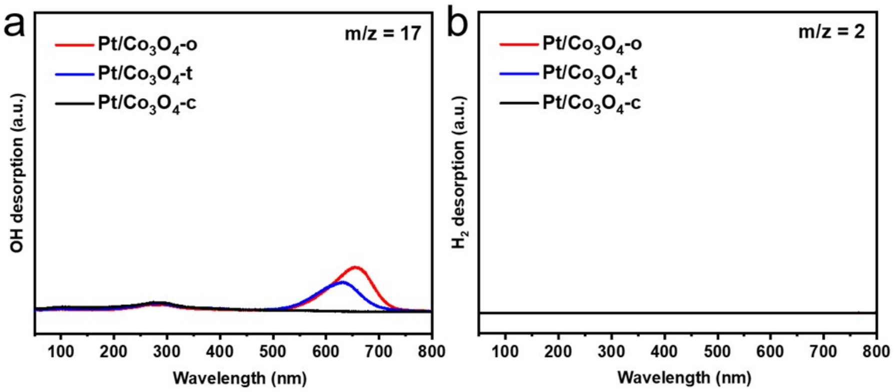

Figure S5. H_2-TPD-MS profiles of the Pt/Co_3O_4-x (x = o, t, c) catalysts: (a) OH species desorption signals (monitored at m/z = 17); (b) H_2 desorption signals (monitored at m/z = 2).

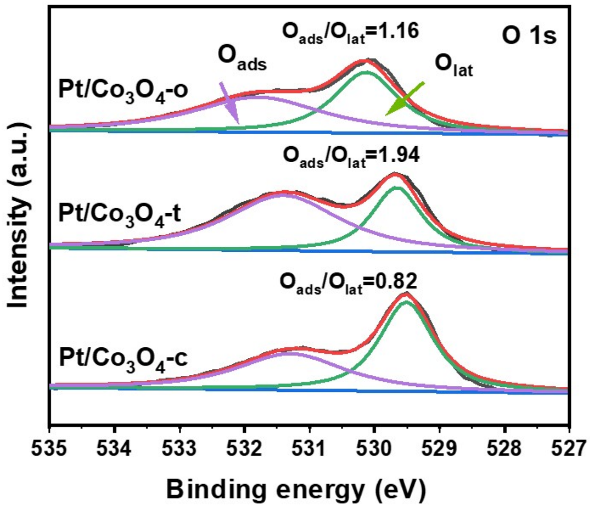

Figure S6. XPS spectra of O 1s for Pt/Co_3O_4-x (x = o, t, c).

The peaks at ~529.9 and 531.7 eV are assigned to the lattice oxygen (O_{lat}) of Co_3O_4 and the absorbed oxygen (O_{ads}) on the oxygen vacancies of Co_3O_4 surfaces, respectively. The higher O_{ads}/O_{lat} area ratio indicates the existence of more oxygen vacancies. The oxygen vacancy concentration follows the order: Pt/Co_3O_4-t > Pt/Co_3O_4-o > Pt/Co_3O_4-c. However, the hydrogen spillover efficiency follows the order: Pt/Co_3O_4-o > Pt/Co_3O_4-t > Pt/Co_3O_4-c (Figure 3c and 3d). There is no direct linear correlation between hydrogen spillover efficiency and oxygen vacancy concentration on the support surface. This indicates that while oxygen vacancies may play a role, they are not the sole or dominant factor governing the hydrogen spillover efficiency in this system.

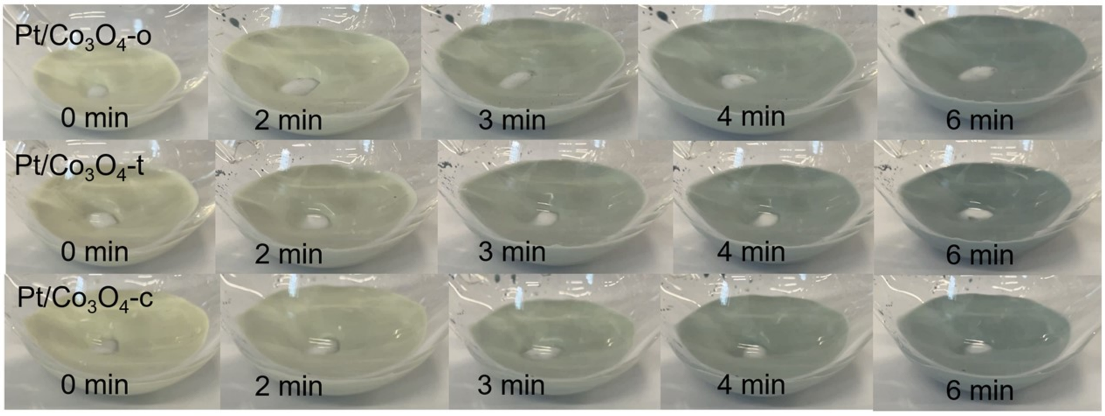

Figure S7. Time-dependent color change of the mixture of WO_3 and Pt/Co_3O_4-x (x = o, t, c) in aqueous phase under H_2 atmosphere.

In this experiment, 1 mg Pt/Co_3O_4-x catalyst was mixed with 300 mg WO_3 in 5 mL water, and H_2 was continuously introduced under stirring. The hydrogen spillover process was visually monitored by the color change of WO_3 (from yellow to blue). The results show that under liquid-phase conditions, all catalysts can induce a distinct color change of WO_3 within a relatively short time, confirming that hydrogen spillover still occurs efficiently in the aqueous environment. This observation is consistent with previous reports on water-promoted hydrogen transfer.[10-12] These findings bridge the gap between gas-phase characterization and liquid-phase reaction environments.

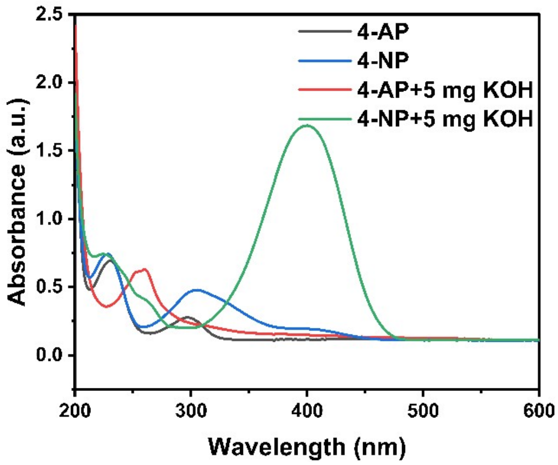

Figure S8. The comparison of UV-vis spectra of 4-nitrophenol (4-NP) and 4-aminobenzenol (4-AP) aqueous solution before and after adding KOH.

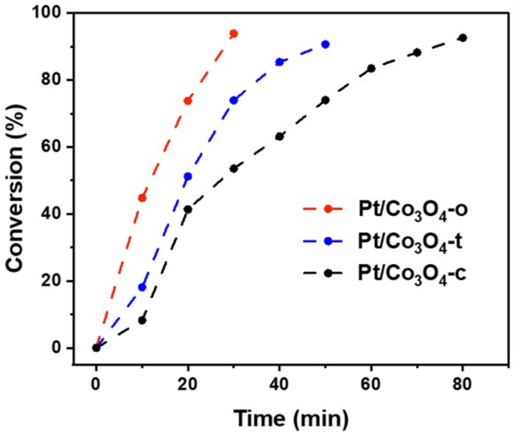

Figure S9. The evolution of 4-NP conversion with the reaction time for Pt/Co_3O_4-x (x = o, t, c).

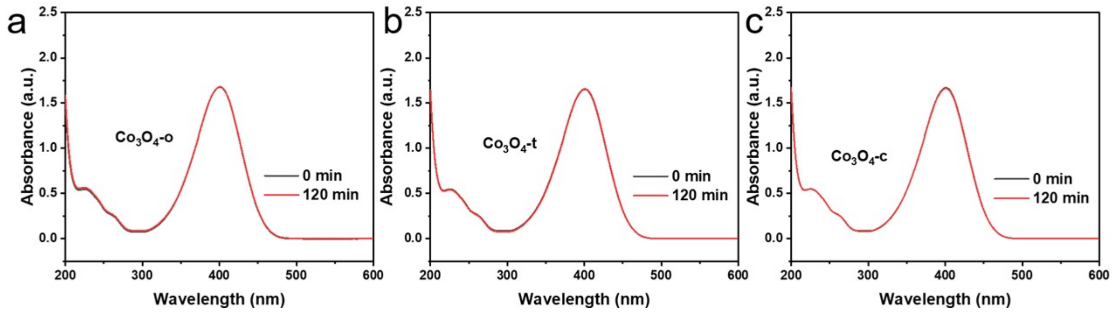

Figure S10. Time-dependent UV-vis spectra of 4-NP reduced by Co_3O_4-o, Co_3O_4-t, and Co_3O_4-c under H_2 atmosphere.

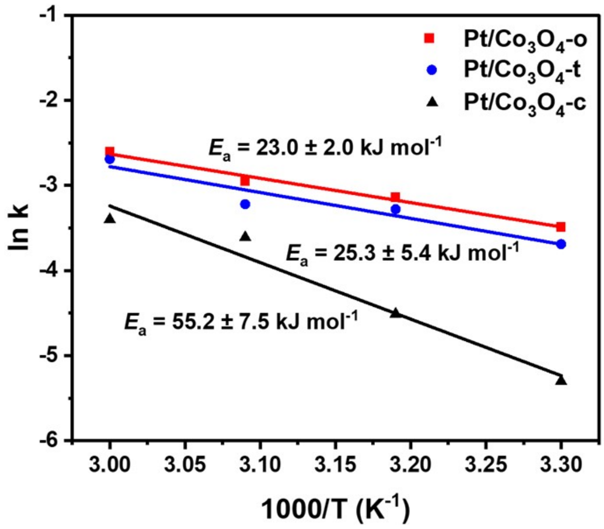

Figure S11. The Arrhenius plots for Pt/Co_3O_4-o, Pt/Co_3O_4-t, and Pt/Co_3O_4.

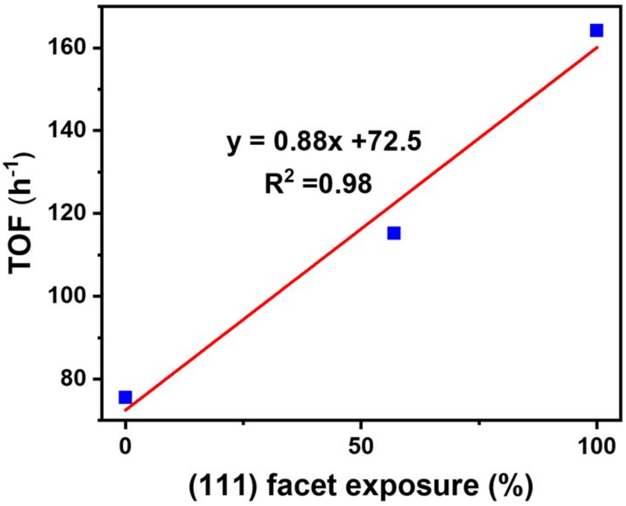

Figure S12. Correlation between (111) facet exposure and turnover frequency (TOF). The intrinsic activity (TOF per Pt site) increases linearly with the percentage of (111) facet exposure across the Pt/Co_3O_4-o, -t, and -c series.

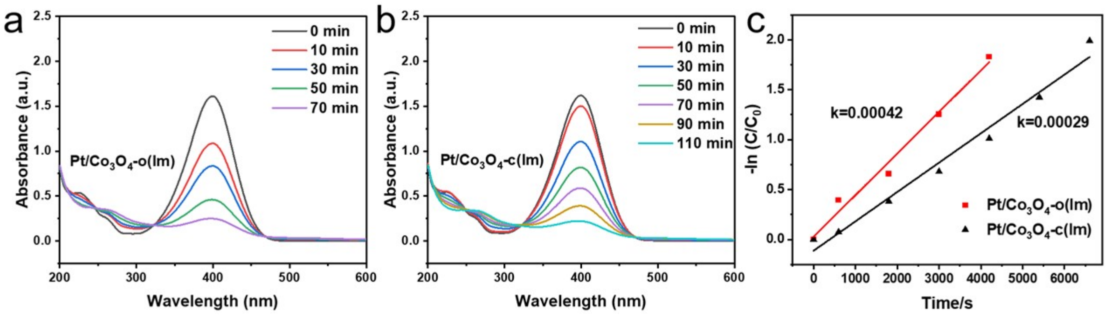

Figure S13. Catalytic hydrogenation of 4-NP over Pt/Co_3O_4 catalysts synthesized via impregnation (Im). Time-dependent UV-Vis spectra for (a) Pt/Co_3O_4-o(Im) and (b) Pt/ Co_3O_4-c(Im). (c) The corresponding pseudo-first-order kinetic plots.

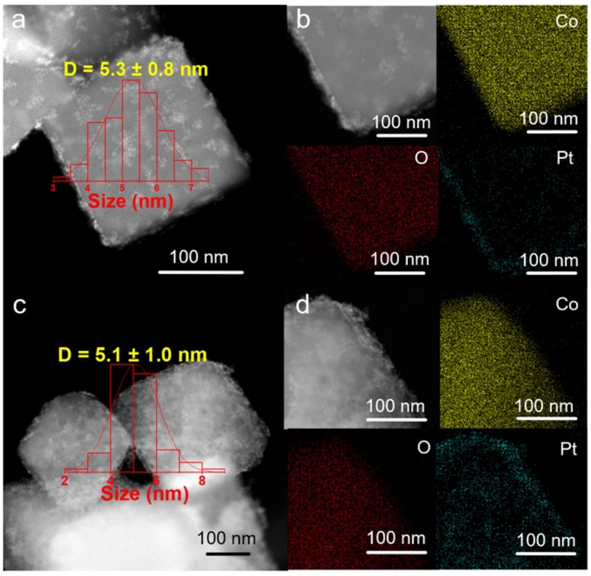

Figure S14. (a, c) HAADF-STEM images and (b, d) STEM-EDS elemental mappings of the impregnation-prepared (a, b) Pt/Co_3O_4-c(Im) and (c, d) Pt/Co_3O_4-o(Im) catalysts.

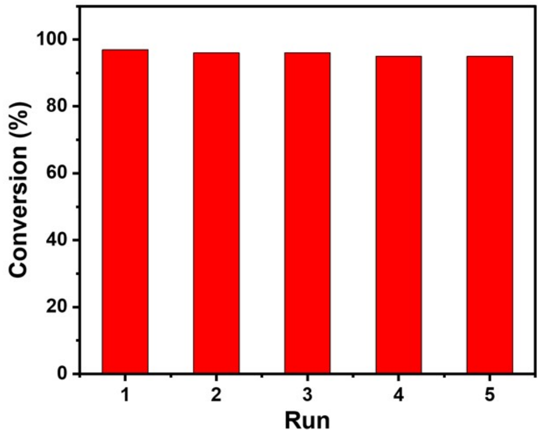

Figure S15. The recycling stability of Pt/Co_3O_4-o for 4-NP hydrogenation.

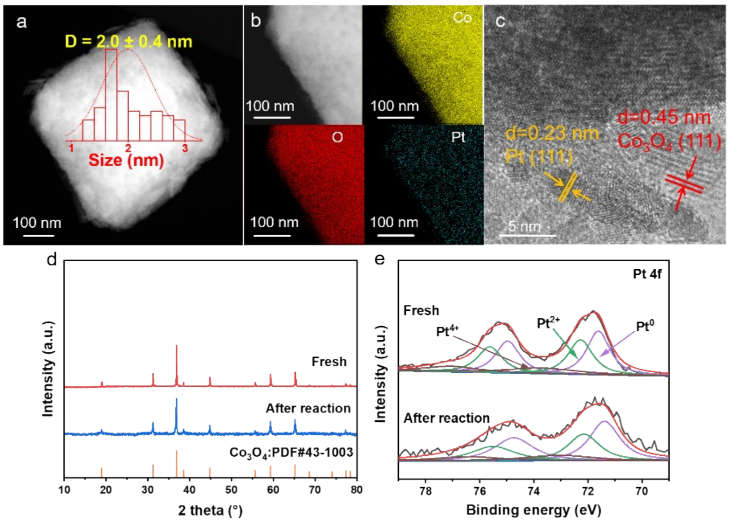

Figure S16. (a) HAADF-STEM, (b) EDS, (c) HRTEM, (d) XRD**,** and (e) XPS images of **t**he Pt/Co_3O_4-o catalyst after five cycles.

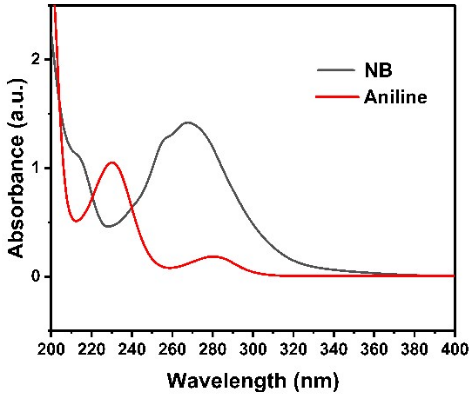

Figure S17. The UV-vis spectra of nitrobenzene (NB) and aniline aqueous solution.

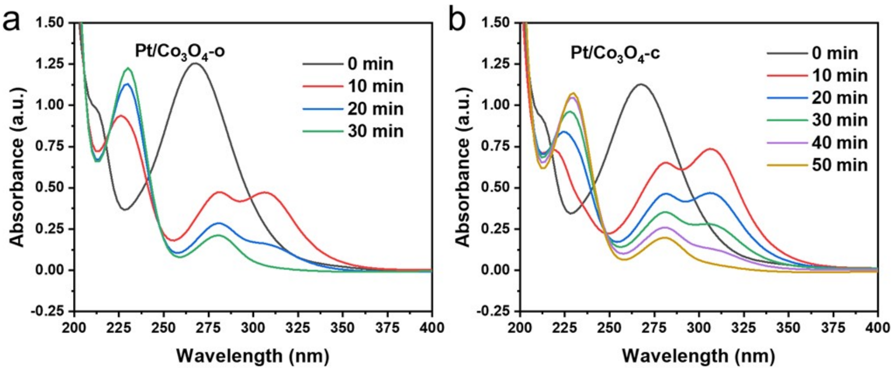

Figure S18. Time-dependent UV-vis spectra of NB reduced by (a) Pt/Co_3O_4-o and (b) Pt/Co_3O_4-c using H_2 as hydrogen source.

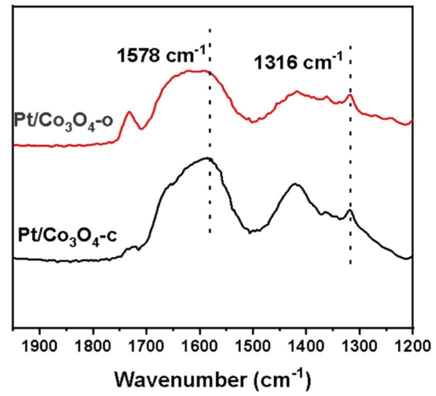

Figure S19. DRIFTS spectra of 4-NP adsorption on Pt/Co_3O_4-o and Pt/Co_3O_4-c.

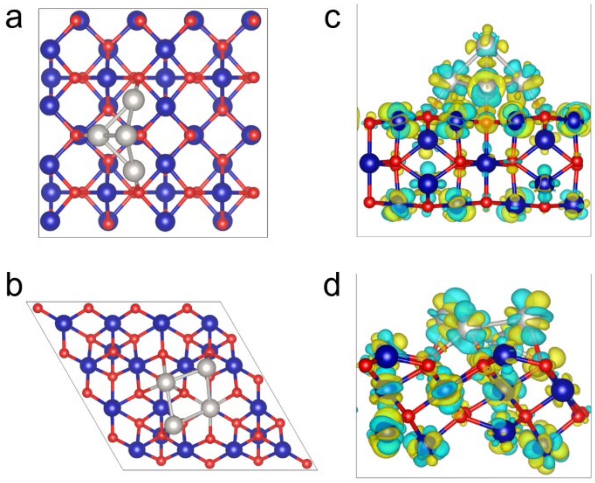

Figure S20. (a, c) Top views and (b, d) charge density difference of Pt_4/Co_3O_4-(100) and Pt_4/Co_3O_4-(111) catalyst models.

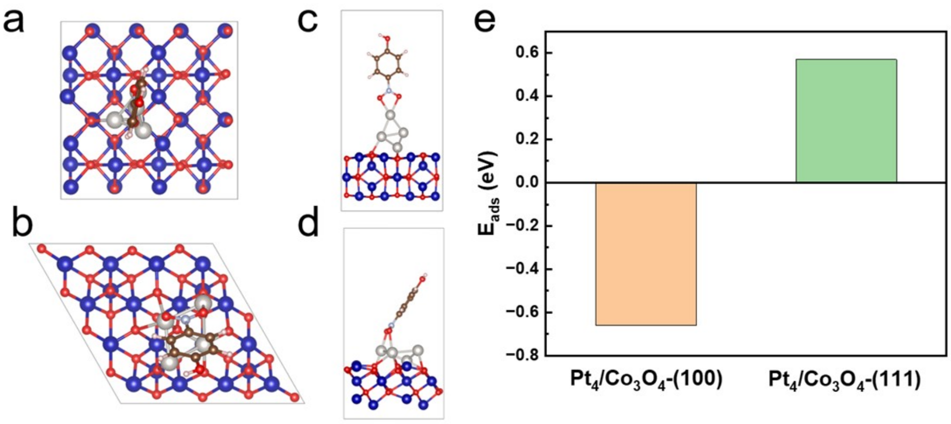

Figure S21. (a-b) Top and (c-d) side views of the adsorption configurations of 4-NP on (a, c) Pt_4/Co_3O_4-(100) and (b, d) Pt_4/Co_3O_4-(111). (e) Adsorption energies of 4-NP on the Pt_4/Co_3O_4-(100) and Pt_4/Co_3O_4-(111) model surfaces.

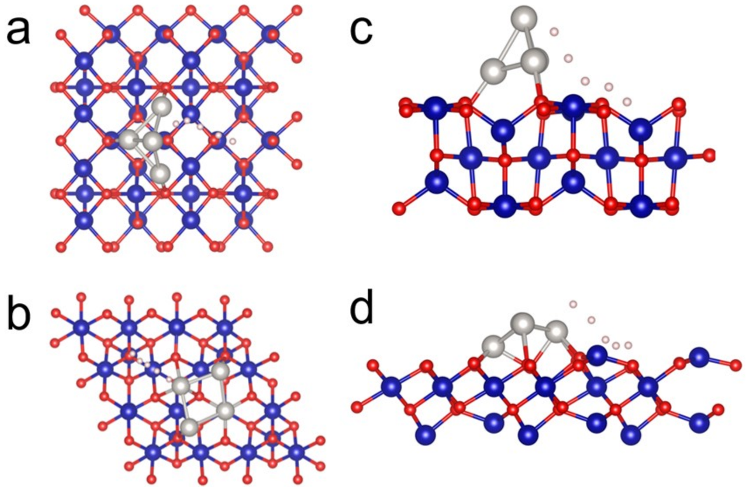

Figure S22. (a-b) Top and (c-d) side views of H-atom migration from Pt_4 to Co_3O_4 surfaces: (a, c) Pt_4/Co_3O_4-(100) and (b, d) Pt_4/Co_3O_4-(111).

Table S1. Loading amounts of Pt and specific surface areas for different catalysts.

Catalysts	Pt contents (wt.%)	BET surface areas（m^2 g^{-1}）
Co_3O_4-o	-	1.7
Co_3O_4-t	-	3.3
Co_3O_4-c	-	3.6
Pt/Co_3O_4-o	0.31%	2.4
Pt/Co_3O_4-t	0.32%	3.4
Pt/Co_3O_4-c	0.31%	3.4

Table S2. Quantitative facet composition of the Pt/Co_3O_4-x (x = o, t, c).

Catalysts	$I_{(111)}/I_{(400)}$[a]	(111) facet exposure (%)[b]
Pt/Co_3O_4-o	0.52	100
Pt/Co_3O_4-t	0.49	57
Pt/Co_3O_4-c	0.45	0

[a] Determined from XRD peak intensity ratios (Figures S3a and c).

[b] We constructed a simple calibration curve using two morphological endpoints: Pt/ Co_3O_4-o (enclosed by (111) facets, assigned as 100% (111)) and Pt/Co_3O_4-c (enclosed by (100) facets, assigned as 0% (111)). Assuming a linear relationship between $I_{(111)}/I_{(400)}$ and the (111) facet abundance, the calculated ratio for Pt/Co_3O_4-t corresponds to a (111) facet exposure of approximately 57%.

Table S3. Comparison of Pt species distribution from XPS analysis for Pt/Co_3O_4-x (x = o, t, c) catalysts under fresh, quasi-in situ (H_2, 40 °C) conditions, and after reaction.

Catalysts	Condition	Pt^0 (%)	Pt^{2+}(%)	Pt^{4+}(%)
Pt/Co_3O_4-o	Fresh	44.7	37.3	18.0
	Quasi-in situ	51.9	29.1	19.0
	After reaction	49.4	34.9	15.7
Pt/Co_3O_4-t	Fresh	39.4	17.8	26.1
	Quasi-in situ	46.7	42.4	10.9
Pt/Co_3O_4-c	Fresh	26.1	51.9	22.0
	Quasi-in situ	40.1	47.7	12.2

Table S4. CO chemisorption uptake and turnover frequency (TOF) of the Pt/Co_3O_4-x (x =o, t, c).

Catalysts	CO uptake[a] ($\mu mol\cdot g^{-1}$)	TOF[b] (h^{-1})
Pt/Co_3O_4-o	8.4	164.2
Pt/Co_3O_4-t	6.5	115.2
Pt/Co_3O_4-c	6.6	75.6

[a] Measured by CO pulse chemisorption at 50 °C.

[b] TOF values were calculated based on the initial 4-NP hydrogenation rate and normalized by the number of surface Pt atoms (from CO uptake, assuming 1:1 CO-to-Pt stoichiometry).

Table S5. ICP-MS analysis of Pt concentration in the post-reaction solution after five cycles and the calculated leaching rate.

Catalyst	Pt in solution (μg L^{-1})[a]	Total Pt loaded (μg)[b]	Leaching Rate (%)[c]
Pt/Co_3O_4-o	1.71 ± 0.02	~60	< 0.2

[a] Average of three measurements.

[b] Calculated based on a 20 mg catalyst charge with 0.31 wt.% Pt loading.

[c] Leaching rate = (Mass of Pt in solution / Total mass of Pt loaded) × 100%.

Table S6. Comparisons of catalytic activities for 4-nitrophenol hydrogenation using H_2 as the reductant over various catalysts.

Catalyst	Reductant	Solvent	T (°C)	P (bar)	K_{app} (s^{-1})	TOF (h^{-1})	Ref.
0.14%Pd/g-C_3N_4	H_2	EtOH	20	20	-	6300	13
2.11%Pd/COF	H_2	EtOH	40	30	-	989.4	14
200O_3-0.91%Pt/CNTs	H_2	H_2O	30	1	-	97.2	15
0.87%Pt NPs@CF	H_2	H_2O	25	1	0.0092	-	16
2Al-2.27%Pt-in-ANTs	H_2	H_2O	25	1	0.0072	-	17
0.083%PdsNC/*PN*-CeO_2	H_2	MeOH	80	5	-	10900	18
Co@NC-1	H_2	EtOH	25	10	-	12.3	19
0.31% Pt/Co_3O_4-o	H_2	H_2O	40	1	0.00152	164.2	This work
0.32% Pt/Co_3O_4-t	H_2	H_2O	40	1	0.00084	115.2	This work
0.31% Pt/Co_3O_4-c	H_2	H_2O	40	1	0.00055	75.6	This work

Table S7. The amount of charge accumulation on Pt_4 in the Pt_4/Co_3O_4-(100) and Pt_4/Co_3O_4-(111) models.

Catalyst models	Bader charge of each Pt atom on Pt_4 (|e|)				Total bader charge on Pt_4 (|e|)
	1	2	3	4	
Pt_4/Co_3O_4-(100)	−0.27	−0.25	−0.40	+0.18	−0.74
Pt_4/Co_3O_4-(111)	−0.80	−0.18	−0.52	−0.15	−1.65

References

1. Q. Sun, X. Wang, H. Wang, H. Zhang, Q. He, Y. Zhang, Y. Cheng, X. Zhang, S. Shi, L. Tao, X. He and H. Ji, Crystal facet effects of platinum single-atom catalysts in hydrolytic dehydrogenation of ammonia borane, *J. Mater. Chem. A.*, 2022, **10**, 10837-10843.
2. Y. Wang, J. W. Ren, K. Deng, L. L. Gui, and Y. Q. Tang, Preparation of tractable platinum, rhodium, and ruthenium nanoclusters with small particle size in organic media, *Chem. Mater.*, 2000, **12**, 1622-1627.
3. G. Kresse and J. Furthmüller, Efficient iterative schemes for ab initio total-energy calculations using a plane-wave basis set, *Phys. Rev. B*, 1996, **54**, 11169–11186.
4. P. E. Blöchl, Projector augmented-wave method, *Phys. Rev. B*, 1994, **50**, 17953–17979.
5. J. P. Perdew, K. Burke and M. Ernzerhof, Generalized gradient approximation made simple, *Phys. Rev. Lett.*, 1996, **77**, 3865–3868.
6. A. Jain, G. Hautier, S. P. Ong, C. J. Moore, C. C. Fischer, K. A. Persson and G. Ceder, Formation enthalpies by mixing GGA and GGA + U calculations, *Phys. Rev. B*, 2011, **84**, 045115.
7. H. J. Monkhorst and J. D. Pack, Special points for Brillouin-zone integrations, *Phys. Rev. B*, 1976, **13**, 5188–5192.
8. D. M. Ceperley and B. J. Alder, Ground state of the electron gas by a stochastic method, *Phys. Rev. Lett.*, 1980, **45**, 566–569.
9. G. Henkelman, B. P. Uberuaga and H. Jónsson, A climbing image nudged elastic band method for finding saddle points and minimum energy paths, *J. Chem. Phys.*, 2000, **113**, 9901–9904.
10. M. Xiong, Z. Gao and Y. Qin, Spillover in Heterogeneous Catalysis: New Insights and Opportunities, ACS Catal., 2021, **11**, 3159-3172.
11. Z. Gu, M. Li, C. Chen, X. Zhang, C. Luo, Y. Yin, R. Su, S. Zhang, Y. Shen, Y. Fu, W. Zhang and F. Huo, Water-assisted hydrogen spillover in Pt nanoparticle-based metal–organic framework composites, Nat. Commun., 2023, **14**, 5836.
12. X. J. Bai, C. Yang and Z. Tang, Enabling long-distance hydrogen spillover in nonreducible metal-organic frameworks for catalytic reaction. Nat Commun, 2024, 15, 6263.
13. L. Q. Li, X. Deng, J. N. He, H. Zhang, L. L and L. H. Zhu, An interfacial synergism effect of Pd–g-C_3N_4 in Pd/g-C_3N_4 for highly active and selective hydrogenation of 4-nitrophenol, *Dalton Trans.,* 2023, **52**, 17974-17980.
14. X. Deng, L. H. Zhu, H. Zhang, L. Q. Li, N. Zhang, J. X. Wang, S. M. Osman, R.

Luque and B. H. Chen, Highly efficient and stable catalysts-covalent organic framework-supported palladium particles for 4-nitrophenol catalytic hydrogenation, *Environ. Res.,* 2022, **214**, 114027.

15. S. F. Xing, Z. Gao, Z. X. Lv, S. C. Zhao, M. Xiong, J. Y. Zhang, G. F. Wang and Y. Qin, Precise regulation of the wettability of Pt/CNTs by atomic layer deposition-based ozone pulse strategy for enhanced catalytic hydrogenation performance in aqueous phase, *Carbon.,* 2022, **188**, 385-392.
16. M. T. Islam, K. A. Sultana and J. C. Noveron, Borohydride-free catalytic reduction of organic pollutants by platinum nanoparticles supported on cellulose fibers, *J. Mol. Liq.,* 2019, **296**, 111988.
17. M. H. Wang, Z. Gao, B. Zhang, H. M. Yang, Y. Qiao, S. Chen, H. B. Ge, J. K. Zhang and Y. Qin, Ultrathin Coating of Confined Pt Nanocatalysts by Atomic Layer Deposition for Enhanced Catalytic Performance in Hydrogenation Reactions, *Chem. Eur. J.,* 2016, **22**, 8438-8443.
18. S. Zhang, C. R. Chang, Z. Q. Huang, J. Li, Z. M. Wu, Y. Y. Ma, Z. Y. Zhang, Y. Wang and Y. Q. Qu, High Catalytic Activity and Chemoselectivity of Sub-Nanometric Pd Clusters on Porous Nanorods of CeO_2 for Hydrogenation of Nitroarenes, *J. Am. Chem. Soc.,* 2016, **138**, 2629-2637.
19. R. J. Gao, L. Pan, Z. W. Li, X. W. Zhang, L. Wang and J. J. Zou, Cobalt nanoparticles encapsulated in nitrogen-doped carbon for room-temperature selective hydrogenation of nitroarenes, *Chin. J. Catal.,* **2018**, 39, 664-672.
